# Hepatic glutamate transport and glutamine synthesis capacities are decreased in finished vs. growing beef steers, concomitant with increased GTRAP3-18 content

**DOI:** 10.1007/s00726-018-2540-8

**Published:** 2018-02-01

**Authors:** J. Huang, Y. Jia, Q. Li, W. R. Burris, P. J. Bridges, J. C. Matthews

**Affiliations:** Department of Animal and Food Sciences, University of Kentucky, Lexington, 40546 USA

**Keywords:** Cattle, Glutamate transport, Glutamine synthetase, Growth phase, Liver

## Abstract

Hepatic glutamate uptake and conversion to glutamine is critical for whole-body N metabolism, but how this process is regulated during growth is poorly described. The hepatic glutamate uptake activities, protein content of system ${\text{X}}^{ - }_{\text{AG}}$ transporters (EAAC1, GLT-1) and regulatory proteins (GTRAP3-18, ARL6IP1), glutamine synthetase (GS) activity and content, and glutathione (GSH) content, were compared in liver tissue of weaned Angus steers randomly assigned (*n* = 8) to predominantly lean (growing) or predominantly lipid (finished) growth regimens. Steers were fed a cotton seed hull-based diet to achieve final body weights of 301 or 576 kg, respectively, at a constant rate of growth. Liver tissue was collected at slaughter and hepatic membranes fractionated. Total (75%), Na^+^-dependent (90%), system ${\text{X}}^{ - }_{\text{AG}}$-dependent (abolished) glutamate uptake activity, and EAAC1 content (36%) in canalicular membrane-enriched vesicles decreased as steers developed from growing (*n* = 6) to finished (*n* = 4) stages, whereas Na^+^-independent uptake did not change. In basolateral membrane-enriched vesicles, total (60%), Na^+^-dependent (60%), and Na^+^-independent (56%) activities decreased, whereas neither system ${\text{X}}^{ - }_{\text{AG}}$-dependent uptake nor protein content changed. EAAC1 protein content in liver homogenates (*n* = 8) decreased in finished vs. growing steers, whereas GTRAP3-18 and ARL6IP1 content increased and GLT-1 content did not change. Concomitantly, hepatic GS activity decreased (32%) as steers fattened, whereas GS and GSH contents did not differ. We conclude that hepatic glutamate uptake and GS synthesis capacities are reduced in livers of finished versus growing beef steers, and that hepatic system ${\text{X}}^{ - }_{\text{AG}}$ transporter activity/EAAC1 content is inversely proportional to GTRAP3-18 content.

## Introduction

Improvement of feeding regimens for production animals has been hindered by a lack of fundamental knowledge about how the capacity to regulate nutrient absorption across cell membranes affects the function of nutrient metabolizing enzymes. Hepatic l-glutamate (Glu) transport and metabolism are critical to support whole-animal energy and N homeostasis (Burrin and Stoll 2009; Heitmann and Bergman 1981; Wu 1998). In the liver, Glu is a central substrate for ureagenesis, gluconeogenesis, glutathione production, de novo protein synthesis, and nitrogen shuttling via glutamine (Meijer et al. 1990; Brosnan 2000; Watford 2000). Although plasma membrane transport capacity is thought to limit Glu metabolism (Gegelashvili et al. 2003; Nissim 1999; Low et al. 1994), knowledge of how hepatic Glu transport capacity may be regulated to support changes in metabolic capacity associated with different phases of growth (e.g., predominately lean to predominately lipid tissue accretion) is limited.

Two high-affinity (μM), concentrative, Glu transporters (GLT-1 and EAAC1) that demonstrate system ${\text{X}}^{ - }_{\text{AG}}$ activity (Na^+^-dependent l-Glu and l-aspartate uptake that is inhibited by d-aspartate) are expressed by the intestinal epithelia, liver, and kidney of sheep (Howell et al. 2001, 2003; Xue et al. 2010) and cattle (Howell et al. 2001; Miles et al. 2015). In mouse neuronal cells, the function of EAAC1 (solute carrier 1A1/SLC1A1) and GLT-1 (SLC1A2) is inhibited by endoplasmic reticulum-localized GTRAP3-18 (glutamate transporter-associated protein 3–18; a.k.a. addicsin, ADP-ribosylation factor-like 6 interacting protein 5/ARL6IP5, PRAF3) (Ruggiero et al. 2008; Watabe et al. 2008) and that the inhibitory effect of GTRAP3-18 on EAAC1 activity is proportional to GTRAP3-18 content (Lin et al. 2001). However, ADP-ribosylation factor-like 6 interacting protein 1 (ARL6IP1) binds and inhibits GTRAP3-18, and indirectly promotes EAAC1 activity (Aoyama and Nakaki 2012, 2013; Akiduki and Ikemoto 2008). The ARL6IP1/GTRAP3-18/EAAC1 triad is highly expressed in rat liver (Akiduki and Ikemoto 2008), whereas cow liver expresses at least EAAC1 and GTRAP3-18 (Miles et al. 2015). The objective of this study was to determine whether the hepatic activities and protein content of system ${\text{X}}^{ - }_{\text{AG}}$ transporters (EAAC1, GLT-1), system ${\text{X}}^{ - }_{\text{AG}}$ regulatory proteins (GTRAP3-18, ARL6IP1), and glutamine synthetase (GS) and glutathione (GSH) contents changed as beef steers developed from predominantly lean (growing) to predominantly lipid (finished) deposition growth phases. The specific hypotheses of this study are that the hepatic activities and protein contents of system ${\text{X}}^{ - }_{\text{AG}}$ transporters and GS, and GSH content, will be (a) increased in finished vs. growing steers and (b) inversely proportional to GTRAP3-18 content, but proportional to ARL6IP1 content in finished vs. growing steers.

## Materials and methods

### Animal model

All procedures involving animals were approved by the University of Kentucky Institutional Animal Care and Use Committee. The steers were raised, and trial conducted, at the University of Kentucky Research and Education Center in Princeton, KY.

Sixteen weaned, predominately Angus, steers were ranked on body weight (BW), and within BW rank, randomly assigned (*n* = 8) to either growing (BW = 215 ± 28.6 kg) or finished (BW = 202 ± 30.2 kg) treatment groups. Steers were then randomly assigned to feedlot pens that contained Calan gates (American Calan, Inc., Northwood, NH) in a dry-lot barn, such that each pen contained 2 steers of each treatment per each of four pens. Steers were individually fed enough of a diet that contained (% as-fed) cracked corn (60), cottonseed hulls (20), soybean meal (7), soybean hulls (5), dried distiller’s grain (2), alfalfa meal (2), glycerin (2), limestone (1.5), and urea (0.5) to support 1.51 kg gain/day (NRC 1996) throughout the trial. Steers had ad libitum access to fresh water and a vitamin-mineral mix (UK IRM Beef Cattle Vitamin-mineral Mix, Burkmann Mill, Inc., Danville, KY). Full BW were determined every 14 days and amount of diet adjusted to achieve target rate of gain. Steer average daily gain (ADG) was calculated as the difference between shrunk (denied feed and water for 14 h) BW at trial initiation and 1 day before slaughter.

### Slaughter, tissue collection, and carcass evaluation

One steer per day was slaughtered. Steers were stunned by captive bolt pistol and then exsanguinated to allow recovery of carcasses for consumption. Fresh liver tissue was collected from the middle of the right lobe and used for (a) preparation of plasma membrane vesicles and determination of glutamine synthesis activity and GSH content, and (b) placed in foil packs, snap-frozen in liquid nitrogen, and stored at − 80 °C until assayed for protein expression.

After 24 h postmortem, carcass evaluations were conducted on the right side of the carcass according to USDA standards (USDA 1997).

### Isolation of enriched canalicular (cLPM) and basolateral (bLPM) liver plasma membrane (LPM) vesicles

Isolation of cLPM- and bLPM-enriched vesicles from liver homogenates was performed as described by Meier and Boyer (1990) with minor modifications. Ten grams of fresh liver tissue was cut into small pieces, washed three times in 80 mL ice-cold 1 mM NaHCO_3_, and then homogenized in 80 mL 1 mM NaHCO_3_ with a Dounce homogenizer (9–11 up-and-down strokes). The homogenate was centrifuged (SA-600 rotor, Sorvall Instruments Inc., Newton, CT) at 1500×*g* for 15 min. The pellet was resuspended in 2.2 volumes of 70% (wt/wt) sucrose. After stirring for 15 min to disrupt membrane aggregates, 12 mL of suspension was filled into SW32 rotor (Beckman Coulter, Inc., Brea, CA) tubes and overlaid with 10 mL 44% and then 10 mL 36.5% (wt/wt) of sucrose. The tubes were filled to the top with 8.1% sucrose and the gradient system centrifuged at 89,300×*g* (SW32 rotor, Beckmann Coulter Inc.) for 90 min. After slow deceleration to a complete stop, a mixed liver plasma membrane fraction was carefully collected from the 44/36.5% sucrose interface with a plastic Pasteur pipet and diluted with 8.1% sucrose to a total volume of 9.1 mL. The mixed liver membrane fraction was then homogenized in a glass–glass Dounce homogenizer using 50 strokes (1 stroke equals up and down) and loaded on top of a three-step sucrose gradient consisting of 10.5 mL 38%, 6.5 mL 34%, and 6.5 mL 31% sucrose. The tubes were centrifuged at 195,700×*g* (SW41 rotor, Beckman Coulter Inc.) for 3 h. This resulted in three distinct bands and a pellet. The fractions at the 31/34% (cLPM-enriched) and the 34/38% (bLPM-enriched) interfaces were recovered, diluted in 8.1% sucrose and sedimented at 105,000×*g* (Ti 70 rotor, Beckman Coulter Inc., Brea, CA) for 60 min. The resulting cLPM and bLPM pellets were resuspended in the preload buffer (250 mM sucrose, 100 mM KCl, 0.2 mM CaCl_2_, 50 mM Hepes/Tris, pH 7.5) by repeated (20 times in and out) aspiration through a 25-gauge needle. The protein content of cLPM and bLPM was quantified at 280 nm using a NANODROP ND-1000 spectrophotometer (NanoDrop Technologies Inc., Wilmington, DE). The vesiculated membranes were stored in liquid nitrogen for Glu uptake assays, and at – 80 °C for enzyme activity and immunoblot analyses.

### Na^+^K^+^-ATPase and γ-glutamyltransferase activity assays

The Na^+^K^+^-ATPase activity in liver homogenate and cLPM and bLPM vesicles was determined using an ATPase assay kit (Sigma, St. Louis, MO) in 96-well plates, as per manufacturer’s instructions. The reaction was initiated by combining 30 μL of pre-incubated (37 °C for 30 min) sample solution (40 mM Tris, 80 mM NaCl, 8 mM MgAc_2_, 1 mM EDTA, pH 7.5) containing 0.35 µg of homogenate or vesicle protein (in the absence or presence of 1 mM ouabain) with 10 μL of 4 mM ATP. After a 30 min incubation period at room temperature, the reaction was terminated by the addition of 200 μL of malachite green reagent. The plate was further incubated at room temperature in the dark for 30 min. The amount of inorganic phosphate (P_i_) released from ATP was quantified colorimetrically at 620 nm (SpectraMax 250, Molecular Devices, Sunnyvale, CA). Samples were assayed in triplicate and Na^+^K^+^-ATPase-specific activity (μmol P_i_ min^−1^ μg^−1^ protein) was calculated by subtracting the ouabain-insensitive activity from the overall activity (in the absence of ouabain). The inter-assay CV and intra-assay CV were 8.5 and 1.8%, respectively.

γ-Glutamyltransferase (GGT) activity in liver homogenate and cLPM and bLPM vesicles was determined using a high-sensitivity colorimetric assay kit (Sigma, St. Louis, MO) following the manufacturer’s instructions. Ten microliters of sample solution (40 mM Tris, 80 mM NaCl, 8 mM MgAc_2_, 1 mM EDTA, pH 7.5) that contained 35 µg of homogenate or vesicle protein was incubated with 90 μL substrate l-γ-glutamyl-*p*-nitroanilide solution at 37 °C in the dark for 18 min. The amount of chromogen *p*-nitroanilide released was quantified colorimetrically at 418 nm (SpectraMax 250), as described (Meister et al. 1981). Samples were assayed in triplicate, and the activity of GGT is reported as μmol *p*-nitroanilide liberated from l-γ-Glutamyl-*p*-nitroanilide per min by 1 μg of protein (μmol *p*-nitroanilide min^−1^ μg^−1^ protein). The inter-assay CV and intra-assay CV were 13.3 and 3.8%, respectively.

### LPM vesicle transport assays

Uptake of Glu by isolated cLPM and bLPM vesicles was conducted using a rapid filtration technique (Ballatori et al. 1986). The previously frozen and preloaded membrane vesicles (see above) were quickly thawed, diluted in preload buffer to the desired protein concentration (50–100 μg of protein in 20 μL), and aspirated 10 times through a 25-gauge needle. Membrane vesicles and appropriate Glu uptake buffers (22.5 µM unlabeled Glu, 195 mM sucrose, 0.2 mM CaCl_2_, 50 mM HEPES, 5 mM MgCl_2_, and 100 mM of either NaCl or choline chloride, at pH 7.5) were separately pre-incubated at 25°C for 10 min. Uptake assays were initiated by addition of 20 μL of membrane vesicles and 10 µCi of l- [3,4-^3^H]Glu ([^3^H]Glu, PerkinElmer, Waltham, MA; specific activity: 47.5 Ci/mmol) to 80 μL of the appropriate Glu uptake buffer. Uptake reactions were performed at 25°C for 10 min. Preliminary experiments determined that Glu uptake was linear and optimal under these conditions (data not shown).

Uptake reactions were terminated by addition of 2 mL of ice-cold stop solution (175 mM sucrose, 0.2 mM CaCl_2_, 5 mM MgCl_2_, 150 mM NaCl, 10 mM HEPES/Tris, pH 7.5), followed by immediate filtration of the uptake reaction/stop solution mix through Millipore HAWP filters (25 mm, 0.45 μm; Millipore Corp., Bedford, MA), using a 1225 sampling vacuum manifold (Millipore Corp.). The uptake tubes were rinsed 2 times with 2 mL of stop solution, filtering the resulting uptake reaction/stop solution mix through the filter after each rinse. Filters were then washed three times more with 2 mL stop solution/wash, and dissolved in 6 mL of liquid scintillation cocktail (SX 25, Fisher Scientific). The radioactivity per filter was measured by liquid scintillation counting (TRI-CARB 2900TR spectrometer, PerkinElmer, Downers Grove, IL). Nonspecific binding of [^3^H]Glu to filters or vesicles was determined by adding 1 mL 4°C stop solution to the [^3^H]Glu uptake mixture before addition to LPM vesicles. The resulting “background” values were subtracted from each experimental Glu uptake observation. Sodium-dependent Glu uptake activity was calculated as the difference between Glu uptake in the presence of NaCl vs. choline chloride. System ${\text{X}}^{ - }_{\text{AG}}$ activity was calculated as the difference between Na^+^-dependent Glu uptake and Na^+^-dependent Glu uptake in the presence of 500 μM of d-aspartate. Samples were assayed in triplicate and all uptake activities are reported as pmol Glu 10 min^−1^ μg^−1^ protein per steer liver preparation.

### Hepatic glutamine synthetase activity analysis

About 400 mg of liver tissue was homogenized (30,000 rpm for 15 s, twice) in five volumes of ice-cold extraction buffer (pH 7.9) containing 50 mM Tris and 2 mM EDTA, using a PowerGen 125 homogenizer (Thermo Fisher Scientific, Waltham, MA). The homogenate was centrifuged at 2000×*g* and 4 °C for 10 min. The derived supernatant was assayed for glutamine synthetase activity (EC 6.3.1.2) using a radiochemical method modified from that of James et al. (1998). A 50 µL aliquot of the supernatant was mixed with 200 µL of reaction medium which consisted of 0.25 µCi l-[1-^14^C]Glu (ARC, Saint Louis, MO; specific activity: 50–60 mCi/mmol), 25 mM unlabeled Glu, 25 mM MgCl_2_, 25 mM NH_4_Cl, 18.75 mM ATP, 12.5 mM phosphocreatine, 4 units creatine kinase, and 62.5 mM imidazole–HCl buffer, at pH 7.6. The mixture was incubated at 37 °C for 20 min, and the reaction was then terminated by adding 1 mL of ice-cold 20 mM imidazole–HCl buffer, at pH 7.5. An aliquot of the incubate (1 mL) was loaded into a 4 mL prefilled (formate resin) ion-exchange column (Bio-Rad, Hercules, CA) previously rinsed by distilled water. The column was washed with 4 mL of distilled water, and the effluent was collected. An aliquot of the effluent (1 mL) was mixed with 4 mL of ScintiSafe Plus 50% cocktail (Thermo Fisher Scientific, Waltham, MA), and the radioactivity was determined by liquid scintillation counting using a TRI-CARB 2900TR spectrometer (PerkinElmer). Assays were conducted in triplicate. Positive control contained 2.5 units of glutamine synthetase from *Escherichia coli* (Sigma, Saint Louis, MO) instead of supernatant from liver tissue. Negative control contained neither supernatant nor glutamine synthetase. Samples were assayed in triplicate and glutamine synthetase activity is expressed as nmol min^−1^ mg^−1^ wet mass. The intra-assay CV and inter-assay CV were 8.5 and 8.8%, respectively.

### Western blot analysis

In general, Western blot analysis of liver tissue and LPM vesicles was performed as previously described by us (Howell et al. 2001; Miles et al. 2015). For liver homogenates, 1 g of liver tissue was homogenized on ice for 30 s (setting 11, POLYTRON, Model PT10/35; Kinematic, Inc., Neuchâtel) in 7.5 mL of 4 °C sample extraction buffer solution (0.25 mM sucrose, 10 mM HEPES–KOH pH 7.5, 1 mM EDTA, and 50 µL of protease inhibitor (Sigma, St. Louis, MO). Protein was quantified by a modified Lowry assay, using bovine serum albumin as a standard (Kilberg 1989). For liver homogenates (30 μg/lane) and LPM (15 μg/lane, protein quantified during preparation), proteins were separated by 12% SDS-PAGE and electrotransferred to a 0.45 µm nitrocellulose membrane (Bio-Rad, Hercules, CA). Blots were stained with Fast-Green (Fisher, Pittsburgh, PA), and the relative amount of stained protein per lane/sample determined by densitometric analyses and recorded as arbitrary units (Miles et al. 2015; Howell et al. 2001).

The relative protein content of EAAC1, GLT-1, GTRAP3-18, GS, and β-catenin in liver homogenates was detected using antibodies as described previously (Brown et al. 2009; Miles et al. 2015, 2016). For ARL6IP1, an antibody raised against the human ortholog (see below) was validated for the detection of cattle ARL6IP1 using a standard pre-hybridization regimen (Xue et al. 2011; data not shown). More specifically, blots were hybridized with 1 μg of IgG anti-human EAAC1 polyclonal antibody (Santa Cruz Biotechnology, Inc., Santa Cruz, CA), 1 μg of IgG anti-rabbit GLT-1 polyclonal antibody (Abcam Inc., Cambridge, MA), 4 μg of IgG anti-human GTRAP3-18 (Abcam Inc., Cambridge, MA), and 5 μg of IgG anti-human ARL6IP1 (Abgent Inc., San Diego, CA), respectively, per mL of blocking solution [1% nonfat dry milk (wt/vol) in 30 mM Tris–Cl, 200 mM NaCl, 0.1% Tween 20 (vol/vol), pH 7.5] for 1.5 h at room temperature with gentle rocking. For β-catenin detection, blots were hybridized with 14.5 μg of IgG anti-chicken β-catenin (Abcam Inc., Cambridge, MA) per mL of blocking solution [1.5% nonfat dry milk [wt/vol] in 30 mM Tris–Cl (pH 7.5), 200 mM NaCl, 0.1% Tween-20] for 1.5 h at room temperature with gentle rocking, whereas GS was detected using 1.25 μg of IgG anti-sheep polyclonal antibody (BD Biosciences, San Jose, CA) per mL of blocking solution [5% nonfat dry milk (wt/vol), 10 mM Tris–Cl (pH 7.5), 100 mM NaCl, 0.1% Tween 20 (vol/vol)] for 1 h at 37 °C with gentle rocking.

All protein-primary antibody-binding reactions were visualized with a chemiluminescence kit (Pierce, Rockford, IL) after hybridization of primary antibodies with horseradish peroxidase-conjugated donkey anti-rabbit IgG (Amersham, Arlington Heights, IL; GLT-1, EAAC1 and ARL6IP1, 1:5000); horseradish peroxidase-conjugated goat anti-mouse IgG (BD Biosciences, San Jose, CA; GS, 1:5000, and β-catenin, 1:10,000); and horseradish peroxidase-conjugated donkey anti-goat IgG (Santa Cruz Biotechnology; GTRAP3–18, 1:5000).

Densitometric analysis of immunoreactive products was performed as described previously (Howell et al. 2003; Xue et al. 2011; Fan et al. 2004). Briefly, after exposure to autoradiographic film (Amersham, Arlington Heights, IL), a digital image of the radiographic bands was recorded and quantified as described (Swanson et al. 2000). Apparent migration weights (M_r_) were calculated by regression of the distance migrated against the M_r_ of a 16–185 kDa standard (Gibco BRL, Grand Island, NY) using the Versadoc imaging system (Bio-Rad) and Quantity One software (Bio-Rad). Band intensities of all observed immunoreactive species (one for GTRAP3-18, ARL6IP1, GS, and β-catenin; two for EAAC1 and GLT-1) within a sample were quantified by densitometry (as described above for Fast-Green stained proteins) and reported as arbitrary units. Densitometric data were then corrected for unequal (≤ 12%) loading, transfer, or both, by normalizing (Miles et al. 2015) the amount of detected protein to relative amounts of Fast-Green-stained proteins (tissue homogenates) or β-catenin (LPM). For β-catenin normalization, anti-EAAC1 and GLT-1 antibodies were stripped from the blots using RESTORE Western Blot Stripping Buffer (Thermo-Scientific, Rockford, IL), per the manufacturer. Digital images were prepared using PowerPoint software (Microsoft, PowerPoint 2003, Bellvue, MA).

### GSH content in liver

Upon collection, liver tissues (0.30–0.35 g) were rinsed in 0.9% (wt, vol) NaCl solution and homogenized in 2.5 mL 5% (wt, vol) ice-cold metaphosphoric acid solution. The homogenates were then centrifuged at 3000×*g*, 4 °C for 10 min. After centrifugation, 100 μL of supernatant was collected and stored at – 80 °C overnight for use the next day.

The glutathione (GSH) concentration in liver was determined using the GSH-400 kit (Oxis International Inc., Beverly Hills, CA), following the manufacturer’s instructions. The standard curve was prepared using 0, 20, 40, 60, and 80 μmol/L GSH. The final absorbance at 400 nm was measured using a Genesys 20 spectrometer (Thermo Electron Corp., Waltham, MA). Samples were assayed in triplicate and values are reported as mg GSH/g wet tissue. The intra-assay CV and inter-assay CV were 1.59 and 3.60%, respectively.

### Statistical analysis

Individual steers were the observational units. Data were evaluated for normality, homoscedasticity, and independence using the PLOTS = diagnostic options in the PROC GLM statement of SAS (SAS Inst. Inc., Cary, NC), and were found to meet ANOVA assumptions. Except for the enzymatic and immunological characterization of bovine cLPM and bLPM vesicles, all data were analyzed in a one-way ANOVA model to test for finished vs. growing treatment effects. For LPM-derived data (*n* = 4–6; Glu transport, relative transporter content), significance was declared when *P* < 0.10, and for all other data (*n* = 8), treatment differences were considered significant at the *α* = 0.05 level. For the enzymatic characterization of cLPM and bLPM fractionation data, enzyme activities among homogenates, cLPM, and bLPM were compared by ANOVA, followed by Fisher’s protected LSD test. For the immunological characterization of cLPM and bLPM fractionation, the relative amount of β-catenin in cLPM and bLPM vesicles was compared by ANOVA, both within and between growing and finished steers. Unequal experimental treatment observations resulted from loss of sample integrity during collection or storage.

## Results

### Animal model

The growth and carcass characteristics of growing and finished steers are presented in Table 1. The growing treatment steers required 57 ± 7 days to reach target weights and 261 ± 12 days were required for finished treatment steers to reach their target weights. As planned, ADG (1.51 vs. 1.45 kg/day), initial BW (215 vs. 202 kg), and frame scores (4.73 vs. 4.61) did not differ (*P* ≥ 0.42) between growing and finished steers. As expected, finished steers had greater (*P* < 0.01) final BW (91%), hot carcass weight (HCW) (107%), ribeye area (44%), 12th rib adipose (220%), marbling score (126%), yield grade (71%), and whole liver wet weight (44%), while the percentage of kidney, pelvic, and heart fat (KPH) tended (*P* = 0.06) to be greater. In contrast, whole liver wet weight/100 kg of final BW was 25% less (*P* < 0.01) in finished vs. growing steers.

**Table 1:** Growth and carcass characteristics of growing vs. finished Angus steers

Item	Treatment		SEM^a^	*P* value
	Growing	Finished		
Growth				
Initial BW (kg)	215	202	23.5	0.42
Final BW (kg)	301	576	28.7	< 0.01
ADG (kg)	1.51	1.45	0.16	0.55
Frame Score	4.73	4.61	0.25	0.76
Carcass				
HCW (kg)	164	339	14.0	< 0.01
Ribeye area (cm^2^)	53.2	76.8	0.80	< 0.01
12th rib adipose (cm^2^)	0.54	1.73	0.06	< 0.01
Marbling score	296	668	67.8	< 0.01
KPH (%)	1.79	2.10	0.10	0.06
Yield grade	2.13	3.65	0.22	< 0.01
Liver (g)	4031	5786	243	< 0.01
Liver, g/100 kg of BW	1341	1005	0.29	< 0.01

Values are means (*n* = 8) and pooled SEM from growing and finished Angus steers

^a^Most conservative error of the mean

### Enzymatic and immunological characterization of cLPM and bLPM vesicles

The enrichment of cLPM and bLPM from liver tissue homogenate was evaluated by comparing the enzyme activity (μmol min^−1^ μg^−1^ protein) of marker proteins in growing steers (Table 2). The bLPM vesicles had 24 times more (*P* < 0.01) Na^+^K^+^ ATPase activity than liver homogenates (1.655 vs. 0.070) and 60% more (*P* < 0.01) than cLPM vesicles, whereas cLPM had 15 times more (*P* < 0.01) Na^+^K^+^ ATPase activity than liver homogenates (1.039 vs. 0.070). In contrast, the cLPM vesicles had 27 times more (*P* < 0.01) GGT activity than liver homogenates (0.054 vs. 0.002) and 93% more (*P* < 0.01) than bLPM, whereas bLPM had 14 times more (*P* < 0.01) GGT activity than liver homogenates.

**Table 2:** Enzymatic characterization of bovine canalicular (cLPM) and basolateral (bLPM) liver plasma membrane vesicles

Enzyme activity^a^	Homogenate	cLPM	bLPM	SEM^b^	*P* value
Na^+^K^+^-ATPase	0.070	1.039*	1.655^#^	0.049	< 0.01
GGT	0.002	0.054*	0.028^#^	0.054	< 0.01

Values are means (*n* = 3) and pooled SEM of marker enzyme activities in canalicular- and basolateral-enriched liver plasma membrane isolated from growing (BW = 301 kg) Angus steers

*P < 0.01 versus homogenate; ^#^*P* < 0.01 versus homogenate and cLPM values

^a^Values are specific activities expressed as μmol product min^−1^ μg^−1^ protein

^b^Most conservative error of the mean

**Fig. 1:**
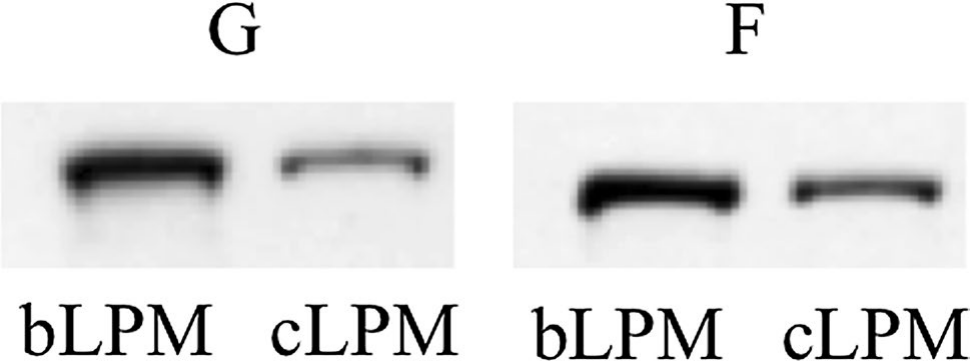
Western blot analysis of β-catenin protein in enriched canalicular- (cLPM) and basolateral- (bLPM) liver plasma membrane vesicles (15 µg per lane) of growing (G) and finished (F) steers. The apparent migration weight of β-catenin was ~ 94 kDa. Data are representative of four growing and four finished steers

To further characterize the membrane composition of cLPM and bLPM vesicles, the relative content of the basolateral marker protein β-catenin (Lutz and Burk 2006; Decaens et al. 2008) was determined by Western blot analysis (Fig. 1) in both growing and finished steers. For growing steers, the β-catenin content of bLPM-enriched vesicles was 158% greater (*P* = 0.003) than for cLPM. Similarly, the β-catenin content of bLPM-enriched vesicles from finished steers was 78.0% greater (*P* = 0.009) than for cLPM. Between growing and finished steers, the amount of β-catenin in cLPM (*P* = 0.75) or bLPM (*P* = 0.88) vesicles did not differ. Consistently, the bLPM:cLPM ratio of β-catenin did not differ (*P* = 0.60) between growing and finished steers.

### Decreased Glu transport activity in hepatic membrane vesicles isolated from finished vs. growing steers

Glu uptake (pmols 10 min^−1^ uptake µg protein^−1^) assays were conducted using canalicular- and basolateral-enriched liver plasma membrane vesicles isolated from livers of growing (*n* = 6) and finished (*n* = 4) steers. In hepatic canalicular plasma membrane vesicles (Table 3), total l-Glu uptake and Na^+^-dependent Glu uptake decreased (*P* ≤ 0.03) 75% and 90%, respectively, whereas Na^+^-independent Glu uptake did not change (*P* = 0.80). Concomitantly, ${\text{X}}^{ - }_{\text{AG}}$ activity (0 ± 0 vs. 2.78 ± 2.57) in canalicular membranes was abolished (*P* = 0.07) and non${\text{X}}^{ - }_{\text{AG}}$ activity tended to decrease (*P* = 0.10) in finished vs. growing steers.

**Table 3:** l-Glutamate (Glu) uptake in canalicular liver plasma membrane vesicles of growing vs. finished Angus steers

Uptake activity^a^	Growing	Finished	SEM^b^	*P* value
Total uptake^c^	8.78	2.23	1.82	0.02
Na^+^-dependent^d^	6.99	0.67	1.89	0.03
Na^+^-dependent (%)^e^	73.2	25.6	11.8	0.01
Na^+^-independent^f^	1.79	1.56	0.65	0.80
Na^+^-independent (%)^g^	26.8	74.4	11.8	0.01
Non${\text{X}}^{ - }_{\text{AG}}$^h^	4.21	0.67	1.19	0.10
Non${\text{X}}^{ - }_{\text{AG}}$ (%)^i^	50.0	100	8.92	< 0.01
${\text{X}}^{ - }_{\text{AG}}$ ^j^	2.78	0	1.05	0.07
${\text{X}}^{ - }_{\text{AG}}$ (%)^k^	50.1	0	8.92	< 0.01

Values are means (*n* = 4–6) and pooled SEM from growing (BW = 301 kg) and finished (BW = 576 kg) Angus steers

^a^Specific uptake activities are expressed as pmols Glu 10 min^−1^ uptake μg^−1^ protein in uptake buffer that contained 25 μM Glu

^b^Most conservative error of the mean

^c^Glu uptake measured in the presence of 100 mM NaCl-containing uptake buffer

^d^Na^+^-dependent Glu uptake is calculated as the difference between total and Na^+^-independent uptake

^e^Percentage of Na^+^-dependent Glu uptake is calculated as the amount of Na^+^-dependent Glu uptake divided by the amount of total Glu uptake

^f^Glu uptake measured in the presence of 100 mM choline chloride-containing uptake buffer

^g^Percentage of Na^+^-independent Glu uptake is calculated as one minus percentage of Na^+^-dependent Glu uptake

^h^Na^+^-dependent Glu uptake that is not inhibited by 500 μM d-Asp

^i^Percentage of non${\text{X}}^{ - }_{\text{AG}}$ activity is calculated as one minus percentage of ${\text{X}}^{ - }_{\text{AG}}$ activity

^j^${\text{X}}^{ - }_{\text{AG}}$ uptake is calculated as the amount of Na^+^-dependent Glu uptake that was inhibited by 500 μM d-Asp

^k^The percentage of ${\text{X}}^{ - }_{\text{AG}}$ activity is calculated as the amount of ${\text{X}}^{ - }_{\text{AG}}$ uptake divided by the amount of Na^+^-dependent Glu uptake

In hepatic bLPM vesicles (Table 4), total l-Glu uptake (56%), Na^+^-dependent Glu uptake (60%), and Na^+^-independent Glu uptake (56%) also decreased (*P* ≤ 0.08) in finished vs. growing steers. However, in contrast to decreased ${\text{X}}^{ - }_{\text{AG}}$ activity in cLPM, ${\text{X}}^{ - }_{\text{AG}}$ activity in bLPM did not change (*P* = 0.76), whereas non${\text{X}}^{ - }_{\text{AG}}$ activity decreased (76%, *P* = 0.06) in finished vs. growing steers.

**Table 4:** l-Glutamate (Glu) uptake in basolateral liver plasma membrane vesicles of growing vs. finished Angus steers

Uptake activity^a^	Growing	Finished	SEM^b^	*P v*alue
Total uptake^c^	5.91	2.39	1.21	0.05
Na^+^- dependent^d^	5.06	2.02	1.22	0.08
Na^+^-dependent (%)^e^	79.8	86.6	6.41	0.47
Na^+^-independent^f^	0.85	0.37	0.17	0.08
Na^+^-independent (%)^g^	20.2	13.5	6.47	0.47
Non${\text{X}}^{ - }_{\text{AG}}$^h^	3.58	0.87	0.93	0.06
Non${\text{X}}^{ - }_{\text{AG}}$ (%)^i^	78.4	37.0	13.0	< 0.01
${\text{X}}^{ - }_{\text{AG}}$ ^j^	1.01	1.15	0.90	0.76
${\text{X}}^{ - }_{\text{AG}}$ (%)^k^	21.6	63.0	13.0	< 0.01

Values are means (*n* = 4–6) and pooled SEM from growing (BW = 301 kg) and finished (BW = 576 kg) Angus steers

^a^Specific uptake activities are expressed as pmols Glu 10 min^−1^ uptake μg^−1^ protein in uptake buffer that contained 25 μM Glu

^b^Most conservative error of the mean

^c^Glu uptake measured in the presence of 100 mM NaCl-containing uptake buffer

^d^Na^+^-dependent Glu uptake is calculated as the difference between total and Na^+^-independent uptake

^e^Percentage of Na^+^-dependent Glu uptake is calculated as the amount of Na^+^-dependent Glu uptake divided by the amount of total Glu uptake

^f^Glu uptake measured in the presence of 100 mM choline chloride-containing uptake buffer

^g^Percentage of Na^+^-independent Glu uptake is calculated as one minus percentage of Na^+^-dependent Glu uptake

^h^Na^+^-dependent Glu uptake that is not inhibited by 500 μM d-Asp

^i^Percentage of non${\text{X}}^{ - }_{\text{AG}}$ activity is calculated as one minus percentage of ${\text{X}}^{ - }_{\text{AG}}$ activity

^j^${\text{X}}^{ - }_{\text{AG}}$ uptake is calculated as the amount of Na^+^-dependent Glu uptake that was inhibited by 500 μM d-Asp

^k^The percentage of ${\text{X}}^{ - }_{\text{AG}}$ activity is calculated as the amount of ${\text{X}}^{ - }_{\text{AG}}$ uptake divided by the amount of Na^+^-dependent Glu uptake

### Decreased EAAC1 protein content concomitant with increased GTRAP3-18 and ARL6IP1 content in liver tissue of finished vs. growing steers

**Fig. 2:**
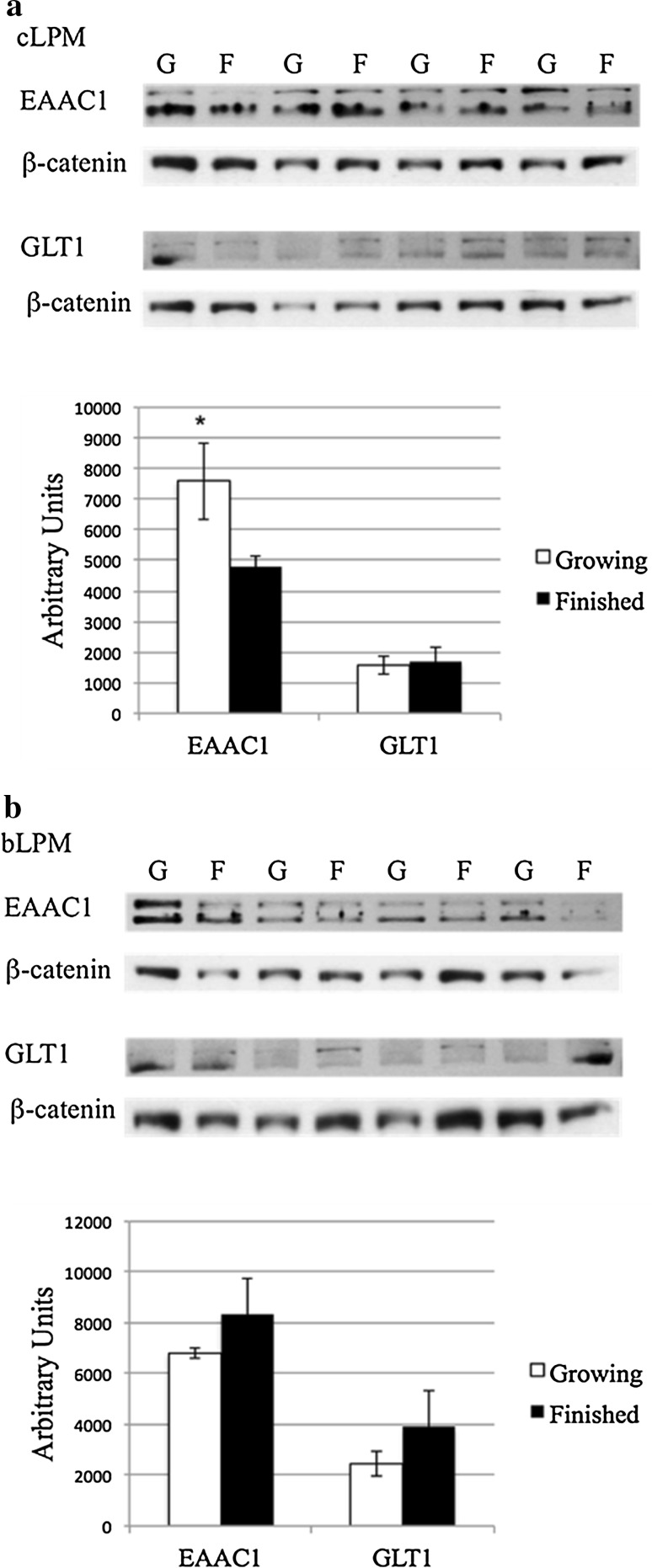
Western blot and densitometric analysis of EAAC1 and GLT-1 in enriched canalicular- (cLPM) (**a**) and basolateral- (bLPM) (**b**) liver plasma membrane vesicles (15 µg per lane) of growing (G, *n* = 4) and finished (F, *n* = 4) steers. The apparent migration weights (kDa) of the lower and upper immunoreactants for EAAC1 were 70 and 89, and 75 and 92 for GLT-1, respectively. Densitometric values reported as means (*n* = 4) ± SE. *Difference at *P* = 0.08 between densitometric values of growing versus finished steers

Canalicular LPM- and bLPM-enriched vesicles were subjected to Western blot analysis (Fig. 2) to determine the relative content of system ${\text{X}}^{ - }_{\text{AG}}$ transporter proteins. Both EAAC1 and GLT-1 were detected in both membrane fractions and development phases. Densitometric analyses found that the amount of EAAC1 decreased (*P* = 0.08) 36% in canalicular liver plasma membrane fraction of finished vs. growing steers (Fig. 2a), but did not differ (*P* = 0.34) in the basolateral fraction (Fig. 2b). However, the phase of growth did not affect (*P* > 0.38) the relative content of GLT-1 in either canalicular (Fig. 2a) or basolateral (Fig. 2b) membrane fractions.

**Fig. 3:**
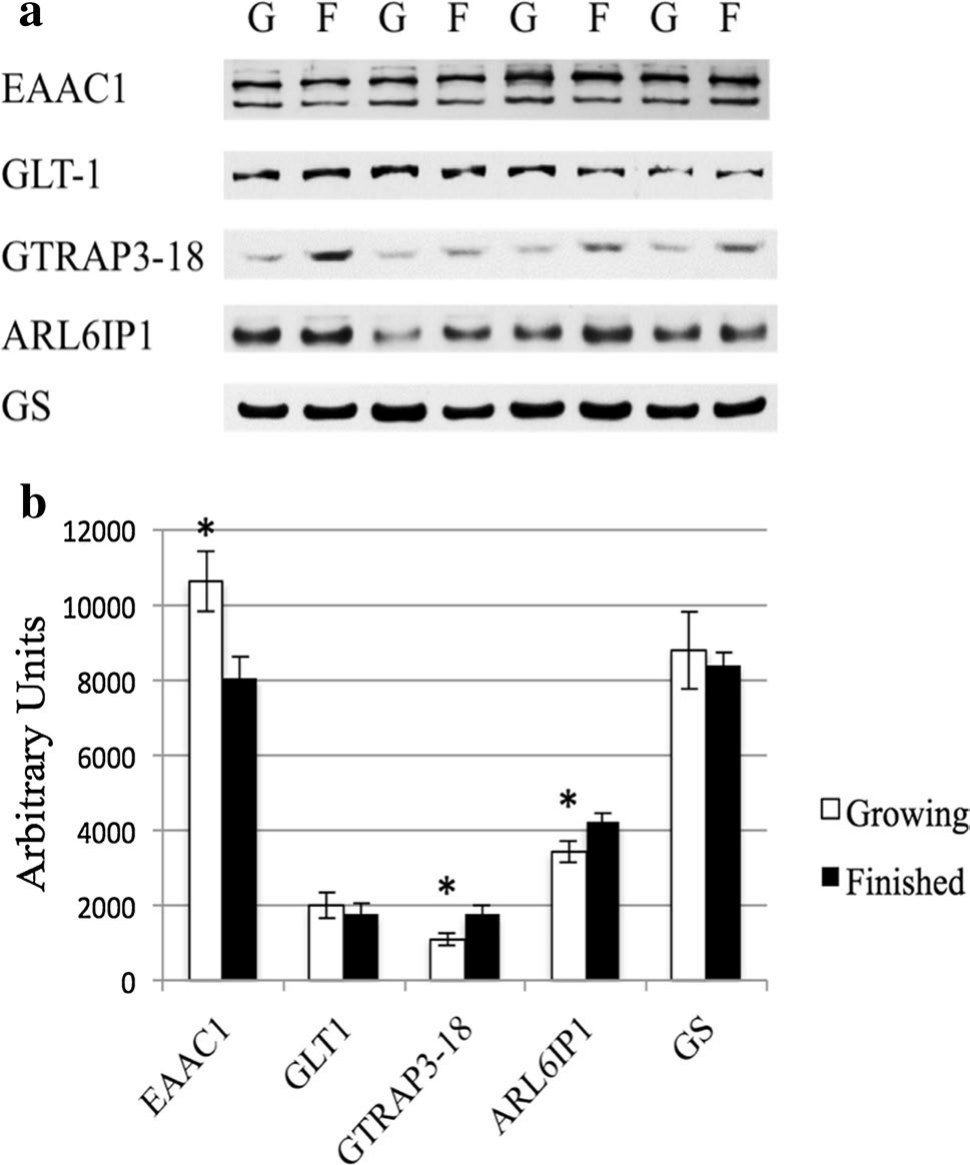
Western blot (**a**) and densitometric (**b**) analysis of EAAC1, GLT-1, GTRAP3-18, ARLI6P1, and glutamine synthetase (GS) in homogenates (15 µg per lane) of growing (G) and finished (F) steers. The apparent migration weights (kDa) for proteins were 70 kDa for the lower, and 89 kDa for the higher, predominant immunoreactants for EAAC1; 75 kDa for GLT-1; 42 for GTRAP3-18; 21 for ARL6IP1; and 43 for GS; respectively. Western blot data are representative of eight growing and eight finished steers. Densitometric values reported as mean (*n* = 8) ± SE. *Difference at *P* ≤ 0.05 between densitometric values of growing versus finished steers

Liver tissue homogenates were subjected to Western blot analysis (Fig. 3a) to determine the relationship among the relative contents of system ${\text{X}}^{ - }_{\text{AG}}$ transporters and their regulatory proteins. All proteins were detected in liver tissue from both development phases. Densitometric analysis (Fig. 3b) found that the relative abundance in liver homogenates of EAAC1 was 24% less (*P* = 0.02) in finished vs. growing steers, whereas GLT-1 content did not differ (*P* = 0.66). Concomitantly, hepatic GTRAP3-18 and ARL6IP1 content increased (*P* ≤ 0.05) 63 and 23%, respectively, in finished vs. growing steers.

### Decreased GS activity, but not GSH content, in liver homogenates of finished vs. growing steers

Glutamine synthetase activity was determined in liver homogenates (Table 5). Paralleling the decreased system ${\text{X}}^{ - }_{\text{AG}}$-specific and total Glu uptake in cLPM vesicles, GS activity decreased (*P* = 0.01) 32% as steers developed from growing to finished production states. In contrast, GS protein content did not differ (*P* = 0.72) between growing and finished steers (Fig. 3).

**Table 5:** Glutamine synthetase (GS) activity and glutathione (GSH) content in liver homogenates of growing vs. finished Angus steers

	Growing	Finished	SEM^a^	*P v*alue
GS activity^b^	0.99	0.67	0.09	0.01
GSH^c^	1.08	1.07	0.04	0.96

Values are means (*n* = 8) and pooled SEM from liver homogenates of growing (BW = 301 kg) and finished (BW = 576 kg) Angus steers

^a^Most conservative error of the mean

^b^Values are nmol min^−1^ mg^−1^ wet tissue

^c^Values are mg GSH/g wet tissue

The GSH content did not differ (*P* = 0.96) between finished and growing steers (Table 5).

## Discussion

A primary function of the mammalian liver is to coordinate whole-body energy and N metabolism. Hepatic transport and intermediary metabolism of Glu is critical to these processes as Glu is a central substrate for hepatic ureagenesis, gluconeogenesis, glutathione production, de novo protein synthesis, and nitrogen shuttling via l-glutamine (Gln) (Meijer et al. 1990; Watford 2000). Understanding how the expression and function of system ${\text{X}}^{ - }_{\text{AG}}$ transporters, their regulatory proteins, and GS activity and GSH content are coordinated could yield important insight into the importance of Glu use and metabolism as production animals grow, especially as they transition from predominantly low to high lipid accretion phases. Accordingly, the objective of this study was to determine if hepatic activities and protein content of EAAC1, GLT-1, GTRAP3-18, and ARL6IP1, and GS activity and protein content, and GSH content, in liver changed as steers developed from growing through finished stages, using a commercially relevant development regimen. To obviate potential metabolic changes as a result of ruminants growing at different rates (Howell et al. 2003), diets for the growing and finished treatment groups were formulated to support the same average rate of growth. As planned, ADG did not differ in finished vs. growing steers and lipid accretion clearly differed among development stages (Table 1). That is, the transition from predominantly lean phenotypes of growing steers to predominantly lipid phenotype of finished steers was demonstrated by higher final BW, HCW, ribeye area, adjusted 12th rib adipose, marbling scores, and yield grade in finished vs. growing steers. Thus, the steers of each treatment group in this study were typical of growing and finished beef cattle phenotypes.

### Characterization of enriched cLPM and bLPM

To gain an insight into the molecular mechanisms of the metabolic function of bovine hepatocytes, it is essential to isolate liver plasma membranes enriched in canalicular and basolateral fractions in sufficient yield to permit functional studies of membrane solute transport processes. Thus, we used a well-established hepatic membrane isolation regimen developed using rat liver (Meier and Boyer 1990) to simultaneously isolate canalicular (cLPM)- and basolateral (bLPM)-enriched plasma membranes from bovine liver.

The enrichment of hepatic canalicular- and basolateral-enriched liver plasma membrane separation was evaluated by determining the relative amount of marker enzyme activities in cLPM and bLPM vesicles vs. the homogenates of liver tissue from which they were isolated (Table 2). Na^+^K^+^-ATPase activity was enriched 22-fold in bLPM and 15-fold in cLPM over homogenates. These results are consistent with the known enrichment of Na^+^K^+^-ATPase activity in both bLPM and cLPM isolated from human and rat hepatocytes (Benkoël et al. 1995; Leffert et al. 1985; Wannagat et al. 1978; Boyer et al. 1983; Schenk and Leffert 1983; Sutherland et al. 1988), and the higher enrichment of Na^+^K^+^-ATPase activity in bLPM than cLPM vesicles (Sutherland et al. 1988; Poupon and Evans 1979; Ali et al. 1990; Boyer et al. 1983).

The enrichment of GGT activity in the cLPM (29-fold) and bLPM (16-fold) vesicles vs. bovine liver homogenates (Table 2) is consistent with the previous findings that GGT is highly enriched in canalicular membranes isolated from rat and human liver (Inoue et al. 1983; Poupon and Evans 1979; Meier et al. 1984) and the 4–19-fold enrichment of the canalicular markers in bLPM isolated from rat liver (Meier et al. 1984). The demonstration of GGT activity in both cLPM and bLPM vesicles isolated from bovine liver is consistent with histochemical studies, showing that GGT is distributed in both canalicular and basolateral membranes of rat and pig liver (Lanca and Israel 1991; Videla and Fernández 1995; Carrion et al. 1993).

The finding that the ratio of β-catenin in bLPM:cLPM did not differ between growing and finished steers (Fig. 1) indicates that the preparation of vesicles was not affected by steer phenotype.

### Glu uptake and Gln synthesis capacity of finished beef steers

An important aspect of Glu metabolism in the liver is the heterogeneity of Gln/Glu metabolism. Specifically, Gln arriving from the peripheral tissues via the portal vein is efficiently absorbed by periportal hepatocytes and deaminated by glutaminase (liver-type) to release ammonia and Glu. The released ammonia can be incorporated into carbamoyl phosphate for ureagenesis, whereas the remaining Glu is available for conversion to α-ketoglutarate (as an anapleurotic reaction to replenish the citric acid cycle), used for gluconeogenesis, used for protein synthesis, or transported into the sinusoids. Whether originating from “up-acinus” periportal hepatocytes, the hepatic artery, or the portal vein, sinusoidal Glu is available for absorption by “down-acinus” pericentral hepatocytes, which express the high-affinity system ${\text{X}}^{ - }_{\text{AG}}$ Glu transporters and Glu-using enzymes for the synthesis of Gln and glutathione (Häussinger and Gerok 1983; Brosnan and Brosnan 2009; Braeuning et al. 2006). If absorbed by pericentral hepatocytes, Glu and the “scavenged” sinusoidal ammonia that escapes incorporation into urea by periportal hepatocytes are incorporated into Gln by pericentral hepatocyte-specific GS activity, thus completing the hepatic Gln-Glu cycle (Moorman et al. 1989, 1990; Wagenaar et al. 1994). Besides supporting Gln synthesis by GS, pericentral hepatocyte-localized system ${\text{X}}^{ - }_{\text{AG}}$ uptake of Glu complements pericentral hepatocyte-specific synthesis of glutathione by γ-glutamylcysteine ligase and glutathione synthetase (Braeuning et al. 2006), as well as supplying Glu as source of gluconeogenic carbons and for de novo protein synthesis (Watford 2000).

Na^+^-dependent, high-affinity Glu transporters are members of the solute carrier family 1A (SLC1A) and function to mediate the Na^+^-dependent, concentrative uptake of Glu, aspartate, and cysteine across cell membranes. The functional capacities of these system ${\text{X}}^{ - }_{\text{AG}}$ transporters are critical to support cell- and tissue-level nitrogen and carbon metabolism (Häussinger et al. 1989; Nissim 1999; Hediger and Welbourne 1999; Heitmann and Bergman 1981; Hundal and Taylor 2009). In bovine liver, EAAC1 and GLT-1 are the only known system ${\text{X}}^{ - }_{\text{AG}}$ transporters (Howell et al. 2001). In rodents, it has been shown that endoplasmic reticulum-localized GTRAP3-18 binds and inhibits the function of EAAC1 and GLT-1 (Ruggiero et al. 2008; Watabe et al. 2008) and that the inhibitory effect of GTRAP3-18 on EAAC1 activity is proportional to GTRAP3-18 content (Lin et al. 2001). GTRAP3-18 is also expressed by bovine liver tissue, and the content of GTRAP3-18 is greater in liver tissue of aged vs. mature cattle, concomitant with decreased GS content (Miles et al. 2015).

To test whether hepatic Glu transport capacity changed as steers develop from growing to finished stages, Glu uptake assays were conducted in cLPM- and bLPM-enriched vesicles from growing and finished steers. Total Glu uptake decreased in canalicular and basolateral plasma membranes as steers developed from growing to finished production stages. This finding may be indicative of a decreased requirement for sinusoidal blood/bile-derived Glu as the steers developed from a predominately lean to predominately lipid tissue accretion phases, due to either (a) an increased supply of intra-cellular Glu from increased glutamate dehydrogenase or ornithine transaminase activity, or (b) a reduced need to capture sinusoidal Glu and ammonia.

Although the activities of glutamate dehydrogenase or ornithine transaminase were not determined, we did measure GS activity in liver homogenates and found that GS activity was decreased 33% in finished vs. growing steers, paralleling the reduced total Glu uptake by both cLPM and bLPM, and system ${\text{X}}^{ - }_{\text{AG}}$-mediated Glu uptake by cLPM. This finding suggests that the ability of liver tissue to use sinusoidal ammonia decreased from growing to finished steers or that the load of sinusoidal ammonia decreased. With regard to the possibility of decreased use of sinusoidal ammonia, detoxification of ammonia by GS may have been limited due to a shortage of intra-cellular Glu (Boon et al. 1999). If so, the above findings suggest that the observed decreased Glu uptake capacity and concomitant decreased GS activity were paralleled by a decreased ability to produce intra-cellular Glu. With regard to the second possibility of decreased sinusoidal levels of ammonia, ammonia concentrations may have been decreased due to an elevated capacity to synthesize urea by periportal hepatocytes.

Regardless of the cause, our findings indicate that the hepatic capacity for plasma membrane Glu uptake and glutamine synthesis was less in liver tissue of finished vs. growing steers. Both of these findings appear incongruent with accepted understandings for nonruminants. That is, for nonruminants, it has been argued that Na^+^-dependent Glu uptake does not limit pericentral hepatocyte GS activity and that GS activity is relatively insensitive to change in various metabolic states (as reviewed by Watford 2000). Thus, our findings may represent another difference in hepatic Gln metabolism between ruminants and nonruminants. For example, during acidosis in nonruminants, the liver switches from a net consumer to net producer of Gln (Heitmann and Bergman 1980), whereas hepatic GS activity and protein content do not change in ruminants (Lobley et al. 2001; Xue et al. 2010).

### Differential expression of EAAC1 transporter in livers of growing and finished steers

As mentioned earlier, EAAC1 is a high-affinity Glu transporter that mediates “system ${\text{X}}^{ - }_{\text{AG}}$” transporter activity. In mouse neuronal cells, EAAC1 activity is regulated by two endoplasmic reticulum (ER)-localized proteins, GTRAP3-18 and ARL6IP1. GTRAP3-18, the negative regulator of EAAC1, decreases EAAC1-mediated Glu uptake by interacting and retaining EAAC1 in the ER, thus delaying EAAC1 trafficking to the trans-Golgi and plasma membrane, increasing its rate of degradation, or both (Lin et al. 2001; Ruggiero et al. 2008). However, an ER localized protein ARL6IP1 serves as a positive regulator of EAAC1 by interacting with GTRAP3-18 to decrease GTRAP3-18/EAAC1 binding. Consequently, high ARL6IP1 expression indirectly promotes the transport activity of EAAC1, at least in mouse neurons (Aoyama and Nakaki 2012; Akiduki and Ikemoto 2008).

In contrast, little is known about the potential regulation of EAAC1 by GTRAP3-18/ARL6IP1 in peripheral tissues. In beef cows, hepatic GTRAP3-18 protein content is greater in aged vs. young cows, whereas EAAC1 and GLT1 contents did not differ (Miles et al. 2015). In the present study with growing vs. finished beef steers, EAAC1 content was less in finished vs. growing steers, concomitant with increased content of GTRAP3-18 and ARL6IP1. Taken together, the decreased system ${\text{X}}^{ - }_{\text{AG}}$-mediated Glu uptake and the reduced EAAC1 protein expression in cLPM in finished vs. growing steers indicate that system ${\text{X}}^{ - }_{\text{AG}}$-mediated Glu transport activity was inversely proportional to GTRAP3-18 content in finished beef steers. Because GS activity in liver homogenates was also decreased in finished vs. growing steers, these findings indicate that a negative functional relationship might exist between GTRAP3-18 content and EAAC1-mediated system ${\text{X}}^{ - }_{\text{AG}}$ and GS activities in bovine liver, as originally identified in rodent neuronal cells (Lin et al. 2001). However, in contrast to ARL6IP1 paralleling EAAC1 content as reported for rat neurons (Akiduki and Ikemoto 2008), ARL6IP1 content was up-regulated 25% in the liver of finished steers, suggesting that either (a) ARL6IP1 content was being stimulated by elevated GTRAP3-18 content, (b) novel regulators might exist in the EAAC1 regulatory network for cattle liver vs. rat brain, or (c) that ARL6IP1 transcription and translation are controlled by discordant regulatory pathways in cattle liver. Overall, additional research is needed using cattle to further investigate pre-translational regulators, including the potential role of microRNAs to regulate expression of EAAC1, GTRAP3-18, and ARL6IP1, as is thought to occur for rodents (Kinoshita et al. 2014).

In addition to Glu and aspartate uptake, system ${\text{X}}^{ - }_{\text{AG}}$ also transports cysteine, the rate-limiting substrate for GSH synthesis (Sato et al. 1999; Matthews 2005; Takada and Bannai 1984; Kilberg 1982). Accordingly, the content of GSH also was measured in the liver. However, GSH content did not differ in the liver of finishing vs. growing steers. Assuming that the intra-cellular GSH synthesis capacity in finishing steers was the same as in growing steers, this finding indicates that hepatic GSH synthesis likely is not limited by EAAC1-mediated cysteine uptake, as evidenced by decreased EAAC1 protein content in liver homogenate and canalicular plasma membrane, and reduced system ${\text{X}}^{ - }_{\text{AG}}$ activity in canalicular liver plasma membrane in finished vs. growing steers. When combined with the higher hepatic GTRAP3-18 content in finished vs. growing steers, this finding is inconsistent with a previous finding in human embryonic kidney 293 cells that GTRAP3-18 negatively regulates cellular GSH content through its interaction with EAAC1 (Watabe et al. 2007) and that suppression of GTRAP3-18 increases neuronal GSH content in GTRAP3-18^−/−^ mice (Aoyama and Nakaki 2012). However, our finding is consistent with the previous studies showing no difference in hepatic GSH level between wild-type and EAAC1^−/−^ mice (Aoyama et al. 2006) and that cysteine in liver is derived from methionine through the trans-sulfuration pathway (Lu 1999, 2013). These results suggest that EAAC1 might play a minor role in delivering substrates for GSH synthesis in bovine liver, thus highlighting the relationship of decreased EAAC1 and GS activity in livers of finished vs. growing steers.

In summary, we reject the original hypotheses that hepatic activities and protein contents of system ${\text{X}}^{ - }_{\text{AG}}$ transporters and GS, and GSH content, would be increased in finished vs. growing steers. Instead, the present study found decreased system ${\text{X}}^{ - }_{\text{AG}}$-mediated Glu uptake and reduced EAAC1 protein expression in cLPM of finished vs. growing steers, concomitant with reduced GS activity. In contrast, system ${\text{X}}^{ - }_{\text{AG}}$ activity and protein content did not differ in the bLPM of finished vs. growing steers, concomitant with no difference in liver GSH content. Although speculative, these findings suggest that system ${\text{X}}^{ - }_{\text{AG}}$ activities in canalicular and basolateral membranes support different hepatic functions.

To our knowledge, this is the first report that (a) system ${\text{X}}^{ - }_{\text{AG}}$ activity and EAAC1 transporter content are inversely proportional to GTRAP3-18 content outside of brain tissue/cultured cells, (b) Glu transport capacity shifts in the liver as cattle develops from lean to lipid phenotypes, and (c) system ${\text{X}}^{ - }_{\text{AG}}$ activity and EAAC1 content of steer hepatic apical membranes are decreased concomitant with decreased hepatic GS activity.
